# Characterization of LncRNA SNHG22 as a protector of NKIRAS2 through miR-4492 binding in osteosarcoma

**DOI:** 10.18632/aging.103849

**Published:** 2020-09-20

**Authors:** Huo-Liang Zheng, Run-Ze Yang, Wen-Ning Xu, Tao Liu, Peng-Bo Chen, Xin-Feng Zheng, Bo Li, Lei-Sheng Jiang, Sheng-Dan Jiang

**Affiliations:** 1 Department of Clinic of Spine Center, Xinhua Hospital, Shanghai Jiaotong, University School of Medicine, Shanghai 200082, China

**Keywords:** OS, SNHG22, miR-4492, NKIRAS2

## Abstract

Many studies have revealed the function of long noncoding RNA (LncRNA) in regulating tumorigenesis of osteosarcoma (OS). As a subgroup of LncRNA, small nucleolar RNA host genes (SNHGs) have emerged as potentially important in OS. According to our recent findings, small nucleolar RNA host gene 22 (SNHG22) plays an important role in inhibiting the growth and metastasis of OS. However, the underlying mechanism of SNHG22 in regulating OS progression remains unknown. In this study, we confirmed that SNHG22 was downregulated in OS, and the overexpression of SNHG22 significantly inhibited OS progression *in vivo* and *in vitro*. Meanwhile, overexpression of SNHG22 also inhibited the migration and proliferation of human umbilical vein endothelial cells (HUVECs) and prevented the epithelial-to-mesenchymal transition (EMT) in OS. Furthermore, the interaction between miR-4492 and SNHG22 we previously predicted was validated by RNA pull-down assays and RNA immunoprecipitation assays. Dual-luciferase reporter assays showed that SNHG22 could directly interact with miR-4492 and upregulate the expression of NK-κB inhibitor-interacting Ras-like 2 (NKIRAS2) by its competing endogenous RNA (ceRNA) activity on miR-4492. In conclusion, our study has clarified the function of SNHG22 in OS progression and suggests a novel therapeutic target for OS.

## INTRODUCTION

Osteosarcoma (OS) is one of the most common primary malignant bone cancers in patients, which presents with two incidence peaks before older adulthood and frequently occurs in long bones such as distal femur and proximal tibia [1]. For its highly aggressive and lung metastatic nature, the 5-year survival rate is only about 19% in OS patients with lung metastasis [1, 2]. Compared with past decades, the therapeutic strategies for treating OS including surgery, chemotherapy and concomitant adjuvant therapy have made great progress, but the prognosis for OS patients with recurrence or metastases is still not optimal [3]. Therefore, it is important and urgent to develop new therapeutic targets for OS patients.

As a type of noncoding RNA, long noncoding RNA (LncRNA) contains more than 200 nucleotides without protein-coding ability [4]. Increasing evidence has shown the high correlation between dysregulation of LncRNA expression and tumor progression involving cell proliferation, invasion and apoptosis [5]. Recently, small nucleolar RNA host genes have become a focus of LncRNA research as potential therapeutic targets of cancer. It has been reported that LncRNA SNHG1 is associated with immune escape of breast cancer [6] and that SNHG8 promotes hepatocellular carcinoma recurrence [7]. SNHG22 has been reported for its impact on epithelial ovarian carcinoma [8]. However, there is little research to our knowledge revealing the functional role of SNHG22 in the development of OS.

In this study, we investigated the impact of SNHG22 on OS and studied the underlying mechanisms. We found that SNHG22 was downregulated in lung metastatic sites compared with orthotopic OS. Meanwhile, we revealed that SNHG22 could not only inhibited OS cell proliferation, migration and invasion, but also induced cell cycle arrest *in vivo* by sponging miR-4492 and acting as a competing endogenous RNA (ceRNA) for NK-κB inhibitor-interacting Ras-like 2 (NKIRAS2). Furthermore, we also showed that SNHG22 suppressed tumor growth *in vitro*. Our research thus indicated that SNHG22 may be a promising therapeutic target in OS.

## RESULTS

### LncRNA SNHG22 is suppressed in OS and associated with tumor metastasis

**Figure 1:**
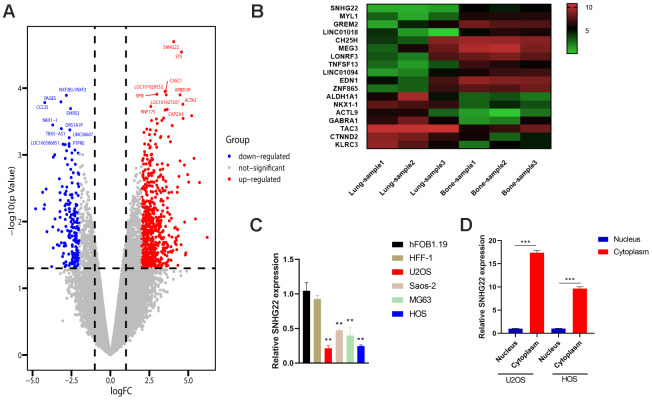
**LncRNA SNHG22 was suppressed in OS and associated with tumor metastasis.** (**A**) Volcano plot of differentially expressed genes in OS and lung metastases. (**B**) Heat map of the associated genes. Red represents upregulation. Green represents downregulation. (**C**) The qRT-PCR confirmed the decreased expression of SNHG22 in OS cell lines. Results are shown as means±SD. n=5; ^**^P<0.01 by *t-*test. (**D**) qRT-PCR analysis of SNHG22 expression in the nucleus and cytoplasm of U2OS or HOS cells. Results are shown as means±SD. n=5; ^***^P<0.001 by *t-*test.

An OS orthotopic lung metastasis model was established by injecting well5 cells into the proximal tibia. Total RNA was extracted from OS cells at the primary site and from lung metastases (GSE85537). Bioinformatics analysis revealed that LncRNA SNHG22 was highly expressed in orthotopic OS cells while it was only weakly expressed in lung metastatic cells (Figure 1A, 1B). However, bioinformatics analysis of GO and GSEA didn’t indicate there were significant changes in cell cycle, EMT pathway between the primary site and lung metastases site (Supplementary Figure 1). qRT-PCR showed that the expression level of SNHG22 was lower in U2OS, MG63, SAOS-2 and HOS cell lines compared to its expression in normal osteoblast cell lines (Figure 1C). Given the unknown role of SNHG22 as a ceRNA, we performed qRT-PCR to measure the expression level of SNHG22 in the nucleus and cytoplasm (Figure 1D). The results showed that SNHG22 expressed more strongly in the cytoplasm.

### SNHG22 induced cell cycle arrest in the G1 phase and inhibited the proliferation, migration and invasion of OS

**Figure 2:**
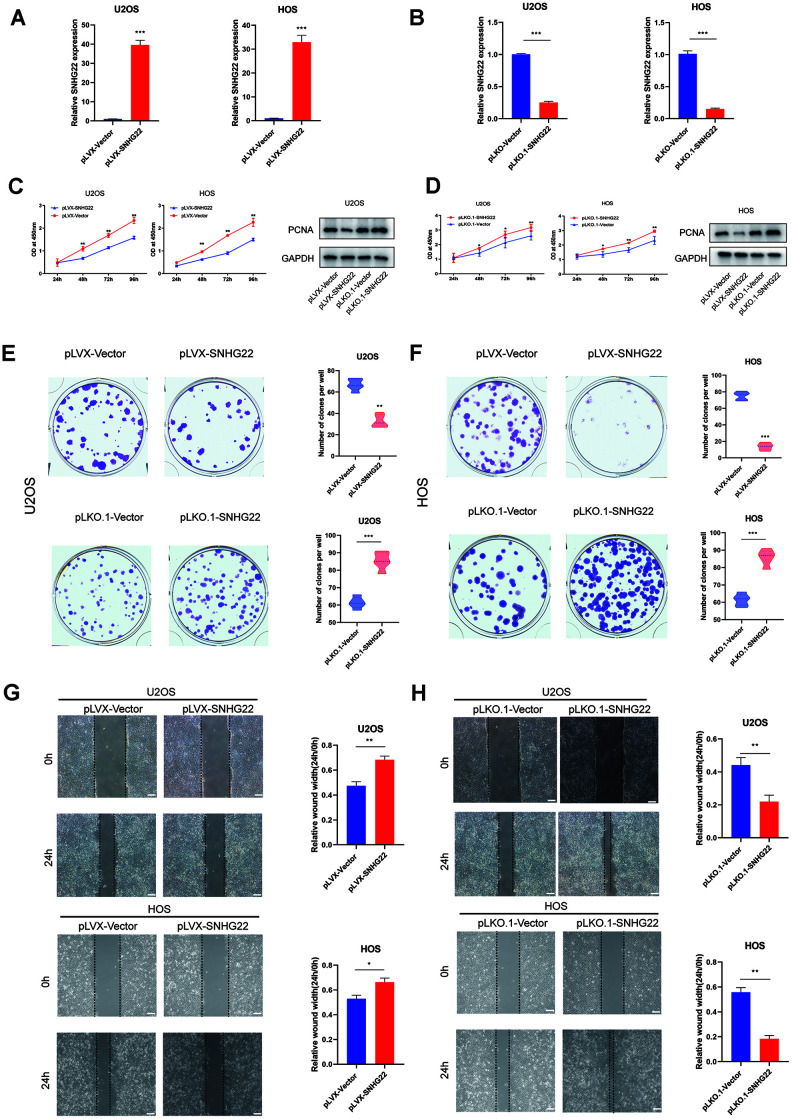
**SNHG22 influenced the proliferation and migration of OS cells.** (**A**, **B**) The qRT-PCR measured the transfection efficiency of SNHG22 in U2OS and HOS cells. n=5; ^***^P<0.001 by *t* test. (**C**, **D**) Overexpression of SNHG22 reduced the cell viability of U2OS and HOS cells while SNHG22 knockdown showed the reverse effect. Cell viability and proliferation were assessed by CCK-8 assays and western blots. n=5; ^*^P<0.05, ^**^P<0.01. (**E**, **F**) Colony formation of HOS and U2OS cell lines was synergistically suppressed by overexpression of SNHG22 and was obviously enhanced by SNHG22 silencing. The number of clones is shown in the violin plot. n=5; ^**^P<0.01, ^***^P<0.001 by *t-*test. (**G**, **H**) Cell migration rates of U2OS and HOS cells were measured by wound healing assays; scale bar: 100 μm. n=5; ^*^P<0.05, ^**^P<0.01, ^***^P<0.001 by *t-*test.

**Figure 3:**
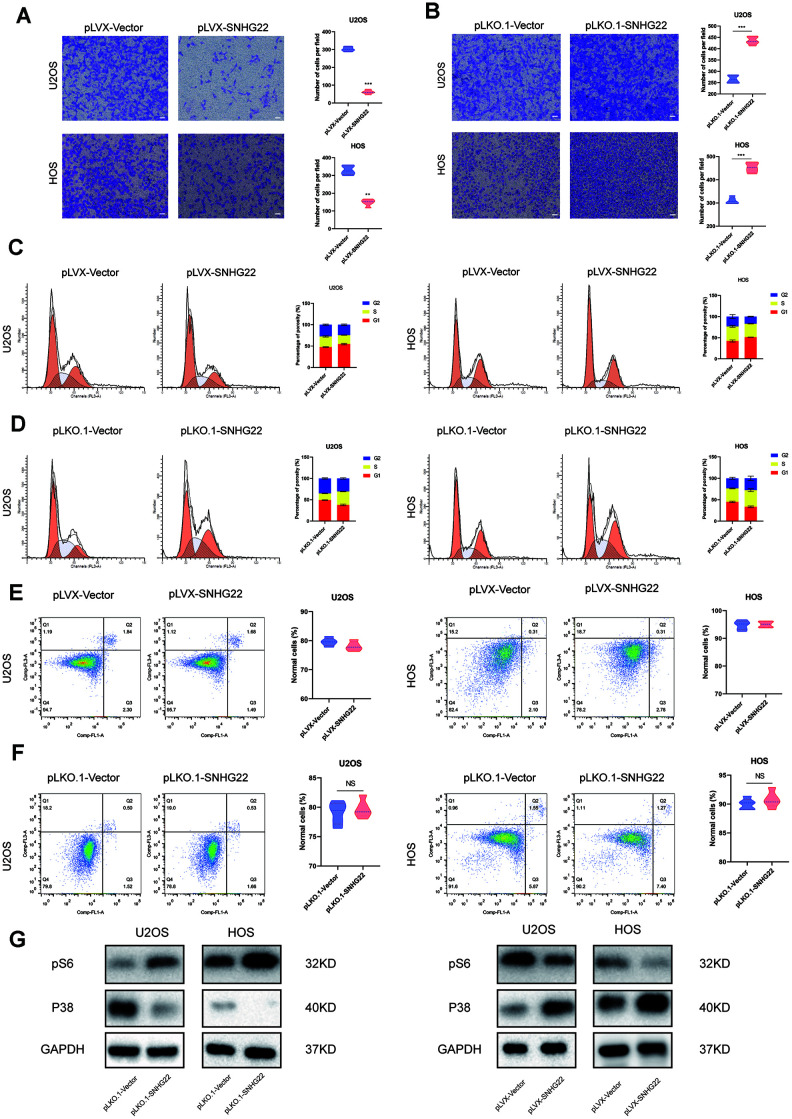
**SNHG22 induced cell cycle arrest and influenced the invasion of OS cells.** (**A**, **B**) Representative images of invading cells in the PLVX-SNHG22, PLVX-VECTOR, PLKO.1-VECTOR and PLKO.1-SNHG22 groups. The number of invading cells is shown in the violin plot. n=5; ^**^P<0.01, ^***^P<0.001 by *t* test. Scale bar: 200 μm. (**C**, **D**) The cell cycle was measured by flow cytometry. The ratio of cells in the G1 phase increased with the overexpression of SNHG22. SNHG22 silencing significantly inhibited cell cycle progression which was arrested in the G1 phase. (**E**, **F**) Apoptosis assays were performed by flow cytometry. Representative images of the ratio of normal OS cells, early apoptotic OS cells, late apoptotic OS cells and dead OS cells are shown. The ratio of normal OS cells was not significantly different. (**G**) Protein expression level of pS6 and p38 was measured by western blot.

We then evaluated the contribution of SNHG22 to the proliferation, migration and invasion of OS. Endogenous SNHG22 levels were silenced or overexpressed by using pLKO.1-SNHG22 or pLVX-SNHG22, respectively, in U2OS and HOS cell lines. The expression level of SNHG22 is shown in Figure 2A, 2B. Cell proliferation was determined by CCK-8 and western blot assays, and the results revealed that SNHG22 knockdown significantly promoted OS cell proliferation (Figure 2D). In contrast, the proliferation of OS cells was greatly impaired when we supplemented exogenous SNHG22 (Figure 2C). We then employed colony formation assays to determine the proliferation capacity of single cells. Notably, colony formation ability in the pLKO.1-SNHG22 group was obviously enhanced compared with the pLKO.1-VECTOR group. We further observed conspicuous inhibition of colony formation in the pLVX-SNHG22 group compared with the pLVX-VECTOR group (Figure 2E, 2F). In addition, wound healing and transwell assays suggested that the migration and invasion capability of U2OS and HOS cell lines was impaired in the pLVX-SNHG22 group compared with the pLVX-VECTOR group. Finally, the suppression of SNHG22 significantly enhanced migration and invasion of the U2OS and HOS cell lines (Figure 2G, 2H and Figure 3A, 3B).

To explore whether the cell cycle and apoptosis were influenced by SNHG22 in the U2OS and HOS cell lines, we performed flow cytometry analysis. Interestingly, we found that compared with the pLKO.1-VECTOR or pLVX-VECTOR group, there was no significant change in the ratio of apoptotic cells in the pLKO.1-SNHG22 or pLVX-SNHG22 group (Figure 3E, 3F). We then investigated whether SNHG22 exerted a regulatory effect on the cell cycle of U2OS and HOS cells. Flow cytometry analysis showed that overexpression of SNHG22 induced cell cycle arrest in the G1 phase. In contrast, the effect was reversed in the pLKO.1-SNHG22 group (Figure 3C, 3D). The protein expression levels of p38 and pS6 indicated that cellular dormancy may be involved in the process of proliferation and migration regulated by LncRNA SNHG22 (Figure 3G).

### SNHG22 inhibited the proliferation and migration of endothelial cells

**Figure 4:**
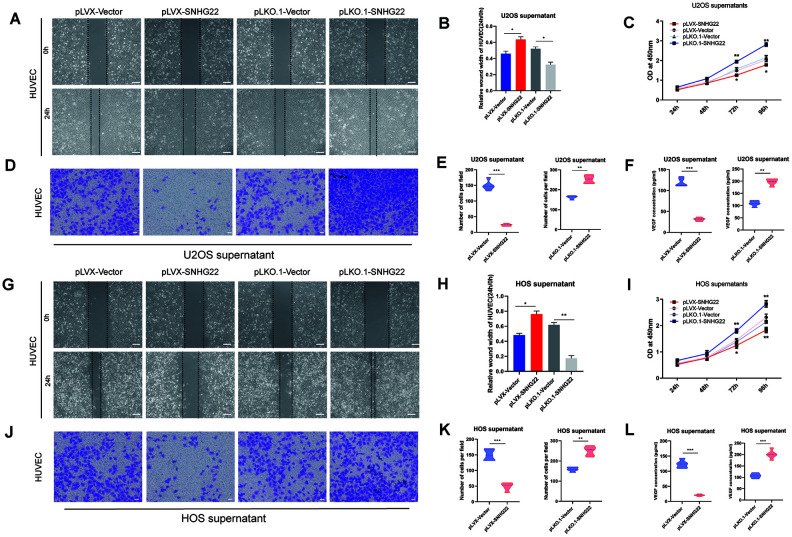
**SNHG22 regulated endothelial cell function.** (**A**) Representative images of HUVEC wound healing assays in the presence of U2OS supernatant. Scale bar: 100 μm. (**B**) Relative cell migration rates of HUVECs were measured. n=5; ^*^P<0.05 by *t-*test. (**C**) Cell viability of HUVECs was assessed by CCK-8 assay. n=5; ^*^P<0.05, ^**^P<0.01. (**D**) Representative images of invading HUVECs. U2OS supernatant was included in all cultures. Scale bar: 200 μm. (**E**) The number of invading HUVECs were counted and are shown in the violin plots. n=5; ^**^P<0.01 by *t-*test. (**F**) The level of VEGF in U2OS supernatant. Results are shown in the violin plots. n=5; ^***^P<0.001 by *t-*test. (**G**) Representative images of HUVEC wound healing assays in the presence of HOS supernatant. Scale bar: 100 μm. (**H**) Relative cell migration rates of HUVECs were measured. n=5; ^*^P<0.05, ^**^P<0.01 by *t-*test. (**I**) Cell viability of HUVECs was assessed by CCK-8 assays. n=5; ^*^P<0.05, ^**^P<0.01. (**J**) Representative images of invading HUVECs. HOS supernatant was included in all cultures. Scale bar: 200 μm. (**K**) The number of invading HUVECs were counted and are shown in the violin plots. n=5; ^**^P<0.01, ^***^P<0.001 by the *t-*test. (**L**) The level of VEGF in HOS supernatant. Results are shown in the violin plots. n=5; ^***^P<0.001 by the *t-*test.

Angiogenesis plays an important role in the tumorigenesis of cancers [9]. To explore whether the migratory ability of endothelial cells was implicated in the anticancer effects caused by overexpression of SNHG22, or in the carcinogenic effect caused by inhibition of SNHG22, we added cell culture supernatants from HOS or U2OS cell lines to human umbilical vein endothelial cells (HUVECs). The cell viability of HUVECs cultured with supernatants from the pLVX-SNHG22 group was impaired as evidenced by CCK-8 assay (Figure 4C, 4I). Wound healing and Transwell assays indicated that the migratory capability of HUVECs cultured with supernatants from the pLVX-SNHG22 group also decreased, while HUVECs cultured with supernatants from the pLKO.1-SNHG22 group showed better migratory capability (Figure 4A, 4D, 4G, 4J). We then asked whether these effects were related to VEGF, so the concentration of VEGF was quantified by ELISA. We found that the concentration of VEGF in the supernatants was obviously decreased in the pLVX-SNHG22 group compared with the pLVX-VECTOR group, while silencing SNHG22 showed the opposite effect (Figure 4F, 4L).

### SNHG22 inhibited the epithelial-to-mesenchymal transition (EMT) of OS

**Figure 5:**
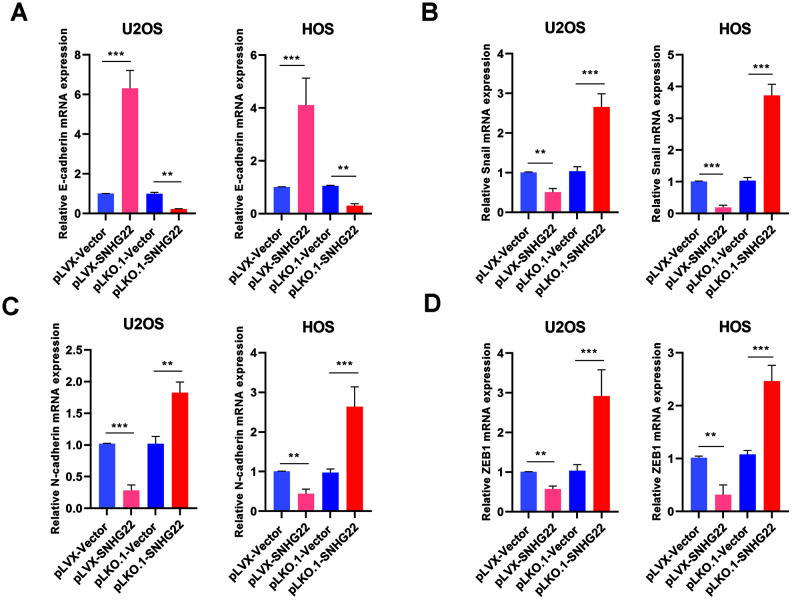
**SNHG22 inhibited EMT in OS.** The expression of EMT-related genes E-cadherin (**A**), snail (**B**), N-cadherin (**C**) and ZEB1 (**D**) in the PLVX-SNHG22, PLVX-VECTOR, PLKO.1-VECTOR and PLKO.1-SNHG22 groups was measured by qRT-PCR and is shown as means±SD. n=5; ^**^P<0.01, ^***^P<0.001 by the *t-*test.

EMT plays an important role in tumor formation and metastasis [10–13]. To characterize the effect of SNHG22 on the EMT of OS, we investigated the expression levels of several representative factors of EMT [14, 15]. The qRT-PCR indicated an increase in mRNA expression of E-cadherin in the pLVX-SNHG22 group compared with the pLVX-VECTOR group, whereas the expression level of E-cadherin was decreased in the pLKO.1-SNHG22 group compared with the pLKO.1-VECTOR group (Figure 5A). Furthermore, other related genes such as Snail, N-cadherin and ZEB1 showed the opposite change (Figure 5B–5D).

### SNHG22 was directly targeted by miR-4492, further regulating the expression of NKIRAS2 through binding with miR-4492

**Figure 6:**
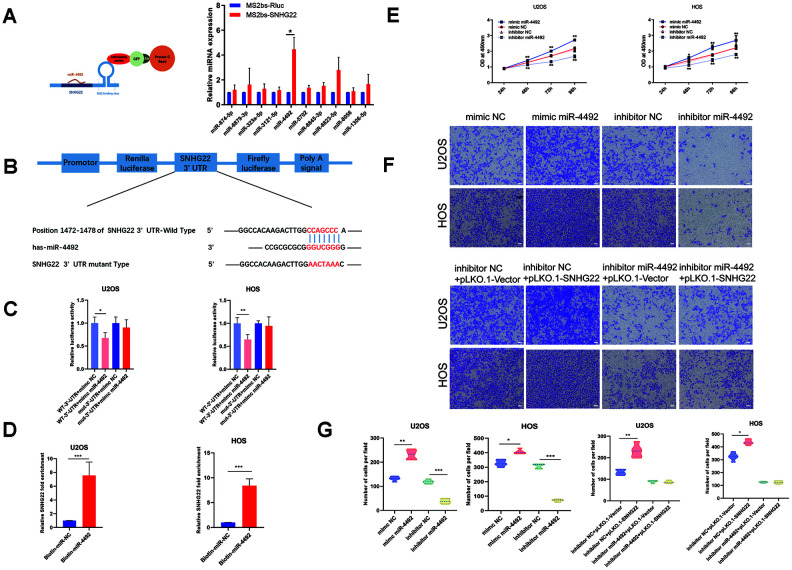
**SNHG22 was associated with miR-4492 in OS cells.** (**A**) Constructs containing SNHG22 transcripts were designed and inserted into MS2bs elements. MS2-RIP was performed, and miR-4492 qPCR was carried out to verify that miR-4492 endogenously associated with SNHG22. n=5; ^*^P<0.05 by *t-*test. (**B**) Plasmids containing mutant or putative SNHG22 3′-UTR-luciferase reporters were transfected into U2OS and HOS cells. (**C**) Luciferase activity in U2OS and HOS cells was detected and is shown as means±SD. n=5; ^*^P<0.05, ^**^P<0.01 by the *t-*test. (**D**) After incubating with biotin-labeled miR-4492, the expression level of SNHG22 was detected by qPCR. n=5; ^***^P<0.001 by the *t-*test. (**E**) Cell viability of U2OS and HOS cells was assessed by CCK-8 assays. n=5; ^*^P<0.05, ^**^P<0.01. (**F**) Representative images of invading cells in mimic NC, mimic miR-4492, inhibitor NC, inhibitor miR-4492, inhibitor NC +PLKO.1-VECTOR, inhibitor NC +PLKO.1-SNHG22, inhibitor miR-4492+PLKO.1-VECTOR and inhibitor miR-4492+PLKO.1-SNHG22 groups. Scale bars: 200 μm. (**G**) The number of invading cells is shown in the violin plot. n=5; ^**^P<0.01 and ^***^P<0.001 by the *t-*test.

Recent studies have revealed that some RNA transcripts can act as ceRNAs by competitively binding miRNAs [16]. To reveal the molecular mechanisms underlying the previously described effects in OS cell lines, we examined the sponging effect of SNHG22. The MS2-bs assay indicated that has-miR-4492 may encompass binding sites for SNHG22 (Figure 6A). The miR-4492 mimic increased the miR-4492 expression level, whereas the miR-4492 level was obviously decreased in OS cells after treatment with miR-4492 inhibitor. We performed dual-luciferase reporter assays and RNA pull-down assays to verify this interaction. Luciferase activity was clearly reduced in the SNHG22-3'-UTR+mimic miR-4492 group compared with the SNHG22-3′-UTR+mimic NC group, while there was no significant difference between the mut-3'-UTR+mimic miR-4492 group and the mut-3′-UTR+mimic NC group (Figure 6C). The interaction between SNHG22 and miR-4492 was further confirmed by RNA pulldown assays using a biotin-labeled specific miR-4492 probe (Figure 6D). Functionally, CCK-8 and Transwell assays suggested that miR-4492 overexpression significantly promoted OS proliferation and invasion, whereas miR-4492 knockdown by specific inhibitor inhibited cell proliferation and migration (Figure 6F, 6G). These results taken together confirmed the interaction between SNHG22 and miR-4492.

**Figure 7:**
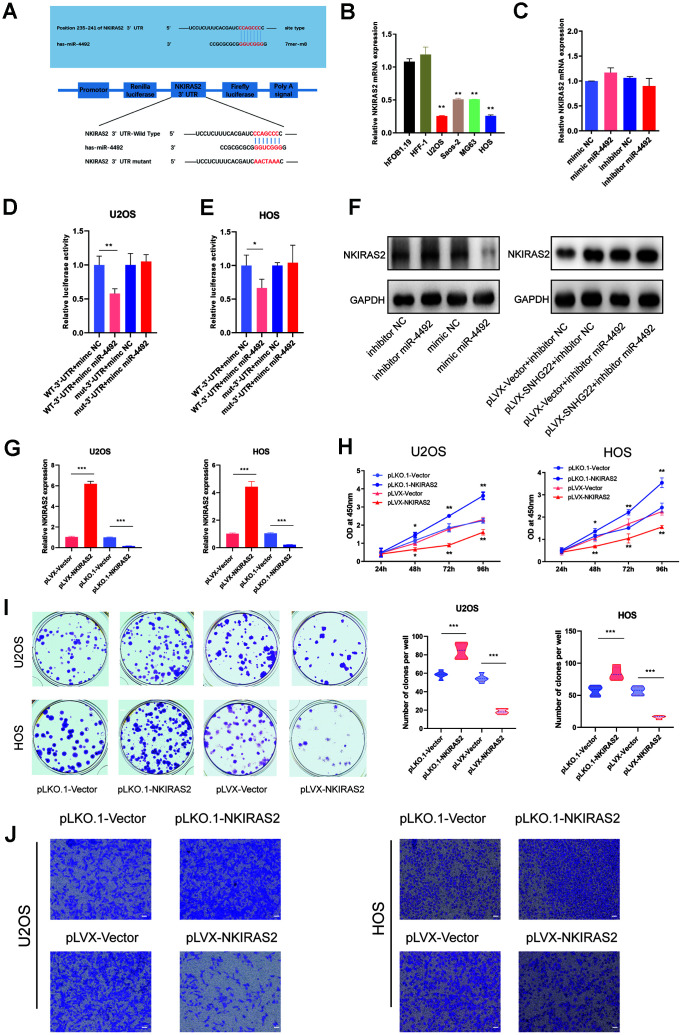
**NKIRAS2 was directly targeted by miR-4492 in OS cells.** (**A**) The miR-4492 was predicted to bind to the NKIRAS2 3′-UTR by Targetscan. Plasmids containing mutant or putative NKIRAS2 3'-UTR-luciferase reporters were transfected into U2OS and HOS cells. (**B**) The qRT-PCR confirmed the decreased expression of NKIRAS2 in OS cell lines. Results are shown as means±SD. n=5; ^**^P<0.01 by the *t-*test. (**C**) The expression of NKIRAS2 in mimic NC, mimic miR-4492, inhibitor NC and inhibitor miR-4492 groups was measured by qRT-PCR. n=5. (**D**, **E**) Luciferase activity in U2OS and HOS cells was detected and is shown as mean±SD. n=5; ^*^P<0.05, ^**^P<0.01 by the *t-*test. (**F**) Proteins were extracted from OS cells and the expression level of NKIRAS2 was analyzed by western blot. (**G**) qRT-PCR measured the transfection efficiency of NKIRAS2 in U2OS and HOS cells. n=5; ^***^P<0.001 by the *t-*test. (**H**) Cell viability of OS cells was assessed by CCK-8 assays. n=5; * P<0.05, **P<0.01. (**I**) Colony formation by HOS and U2OS cell lines was inhibited by the overexpression of NKIRAS2 and was obviously enhanced by NKIRAS2 silencing. The number of clones was shown in the violin plot. n=5; ^**^P<0.01, ^***^P<0.001 by the *t-*test. (**J**) Representative images of invading OS cells. Scale bar: 200 μm.

To determine the underlying mechanisms of miR-4492 in OS, we used two bioinformatics tools, Targetscan and miRanda, to predict the potential downstream targets of miR-4492. The results indicated that NKIRAS2 may be the direct downstream target of miR-4492 (Figure 7A). We hypothesized that miR-4492 promoted the proliferation and migration of U2OS and HOS cell lines through the suppression of NKIRAS2. To verify this, we examined NKIRAS2 mRNA expression levels in OS cells and observed its reduction in OS cells while remaining highly expressed in normal osteoblast cell lines (Figure 7B). Furthermore, we transfected luciferase reporter constructs with the putative or mutated NKIRAS2 3'-UTR into U2OS and HOS cells to study whether NKIRAS2 was directly targeted by miR-4492. miR-4492 overexpression impacted NKIRAS2 3'-UTR reporter activity but had no obvious effect on luciferase activity when the binding sites were mutated in U2OS and HOS cells (Figure 7D, 7E). However, we found that whether or not we transfected mimic miR-4492 or inhibitor miR-4492, qPCR assays suggested the expression level of NKIRAS2 mRNA was not significantly changed (Figure 7C). Consistent with the luciferase results, western blot analysis showed that miR-4492 downregulated the expression of NKIRAS2 while the inhibition of miR-4492 upregulated the expression of NKIRAS2 (Figure 7F). We then used CCK-8 and colony formation assays to reveal that NKIRAS2 knockdown promoted the proliferation of OS cells while the overexpression of NKIRAS2 inhibited their proliferation (Figure 7H, 7I). Furthermore, Transwell assays showed that the invasive capacity of OS cells was significantly inhibited by NKIRAS2 (Figure 7J).

### SNHG22 inhibited tumor growth *in vivo*

**Figure 8:**
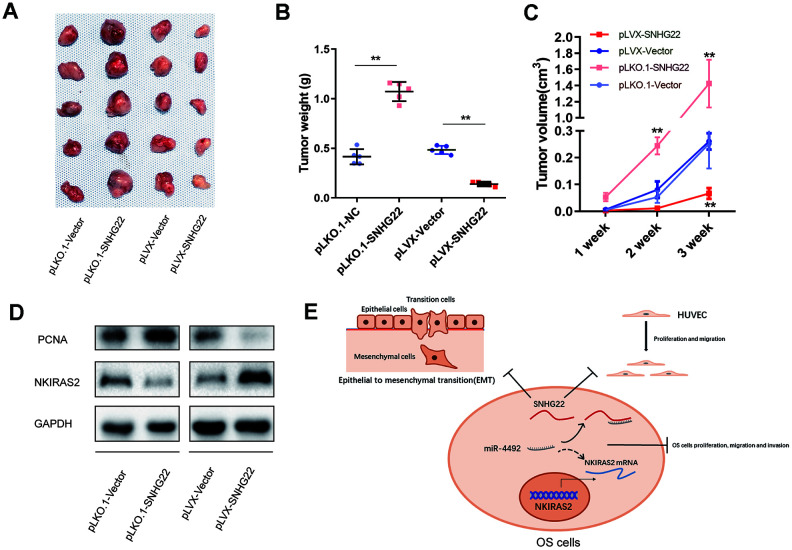
**SNHG22 inhibited tumor growth *in vivo*.** (**A**) HOS cells transfected with PLKO.1-VECTOR, PLKO.1-SNHG22, PLVX-VECTOR or PLVX-SNHG22 were inoculated into nude mice (n=5). The tumors were excised 3 weeks later. (**B**) Tumors from different groups were weighed. ^**^P<0.01. (**C**) Tumor volumes were estimated (0.5 × width^2^ × length) by caliper every week. ^**^P<0.01. (**D**) Proteins were extracted from tumors and the expression level of NKIRAS2 and PCNA was measured by western blot. (**E**) Schematic illustration of the role of SNHG22 in OS cells.

Based on previous observations, the influence of SNHG22 on tumor growth *in vivo* was analyzed in tumor-bearing nude mice. HOS cells transfected with pLKO.1-VEXTOR, pLKO.1-SNHG22, pLVX-VECTOR, or pLVX-SNHG22 were subcutaneously injected into BALB/c nude mice. As shown in Figure 8A, tumors formed in the pLKO.1-SNHG22 group grew faster compared with the pLKO.1-VEXTOR group, while tumors in the pLVX-SNHG22 group grew more slowly compared with the pLVX-VECTOR group. The volumes and weights of tumors were consistent with the above observations (Figure 8B, 8C). The expression level of NKIRAS2 was decreased while the cell proliferation marker PCNA was increased in the pLKO.1-SNHG22 group compared with the pLKO.1-VECTOR, as evidenced by western blot (Figure 8D).

## DISCUSSION

OS is common primary malignant bone cancer and is considered an age-related disease that mainly occurs in patients before older adulthood [17, 18]. It has recently been found that LncRNA is associated with tumor progression which has fueled the continuing interest in finding a novel therapeutic target for OS [18–21]. SNHGs exert their functions through interacting with miRNAs and the downstream pathways mediated by their targets. As a sponge of miRNAs, SNHG ceRNA can regulate miRNA target mRNAs [7]. The more miRNAs bind to the complementary region in SNHGs, the less they bind to the complementary region in target mRNAs. The identification of the SNHG-miRNA-mRNA axis in OS may be helpful in revealing the underlying molecular mechanisms mediated by SNHGs. The miRNAs interacting with SNHGs could differ in different tissue types, probably the result of different gene expression levels under different conditions. For example, Di et al. found that LncRNA SNHG14 facilitates colorectal cancer metastasis through targeting EZH2-regulated EPHA7 [22], and Deng et al. reported that LncRNA SNHG14 potentiates pancreatic cancer progression via modulation of annexin A2 expression by acting as a ceRNA for miR-613 [23]. Although it has been reported that SNHG22 overexpression indicates poor prognosis and induces chemotherapy resistance via the miR-2467/Gal-1 signaling pathway in epithelial ovarian carcinoma [8], the function of SNHG22 in OS remains unknown.

In this study, we demonstrated that SNHG22 is a previously unrecognized regulator of OS progression. We analyzed the GEO dataset (GSE85537) and found that SNHG22 was downregulated in orthotopic OS compared with lung metastatic sites. Cell cytoplasm/nuclear fraction isolation assays followed by qRT-PCR indicated that SNHG22 was mainly localized to the cytoplasm where it can act as a sponge of miRNAs. In our research, we first found that overexpression of SNHG22 induced cell cycle arrest and inhibited the proliferation, migration and invasion of OS cells, while downregulation of SNHG22 played a facilitative role in cell viability and other cell phenotypes.

EMT is characterized by the conversion of apical–basal polarized epithelial cells to mesenchymal spindle-shaped phenotype cells [24, 25]. EMT plays an important role in tumor progression. Because of its crucial role in tumorigenesis, therapy targeting EMT has become more promising [26]. Thus, we investigated if SNHG22 could affect the EMT of OS. As is well known, the upregulation of N-cadherin and the downregulation of E-cadherin is a marker of EMT [27–30]. The expression level of E-cadherin, N-cadherin, Snail and ZEB1 in our experiments was measured by qRT-PCR, which confirmed the link between SNHG22 and EMT of OS.

Due to its essential role as a highway of oxygen and nutrition in tumor progression, angiogenesis is an important therapeutic target for inhibiting tumorigenesis and tumor progression [31]. VEGF expression correlates with angiogenesis and induces endothelial cells to proliferate, migrate and form new vessels [32–34]. Therefore, we studied the impact of SNHG22 on HUVECs. In this case, we showed that the concentration of VEGF in supernatants from the pLVX-SNHG22 group was reduced. However, it should still be mentioned that we cannot exclude effects on HUVECs caused by other factors. Given the role of VEGF in angiogenesis crosstalk, SNHG22 may inhibit HUVEC proliferation and migration by controlling VEGF.

In the course of probing the molecular mechanisms by which SNHG22 regulates OS cells, miR-4492 was found by bioinformatics prediction. Our studies confirmed that miR-4492 specifically bound to SNHG22 by RIP and RNA pull-down assays. It has been reported that miR-4492 is involved in colorectal cancer cell proliferation and bladder cancer progression, but miR-4492 has never been examined in OS cells to the best of our knowledge. In this study, we report that miR-4492 rapidly promoted OS cell viability and invasion and counteracted the SNHG22-induced tumor-inhibiting effect. However, the miR-4492 target mRNA in OS had not previously been identified. We found that NKIRAS2 was an important target of miR-4492 in OS cells with the help of TargetScan.

NKIRAS2, also known as NK-κB inhibitor-interacting Ras-like 2, is involved in the degradation of inhibitor of NF-κB β (IκB-β) and negative regulation of the NK-κB signaling pathway [35]. NKIRAS2 has been reported to mediate nasopharyngeal carcinoma cell growth and metastasis [36]. Haemmig et al. have reported that downregulation of NKIRAS2 promotes the activity of the NK-κB signaling pathway in glioblastomas [37]. In addition, others have also reported that the NF-κB signaling pathway is activated and related to cell apoptosis in OS [38–41]. In this study, we demonstrated that NKIRAS2 expression was downregulated in OS cell lines. The interaction between miR-4492 and NKIRAS2 was then confirmed by a dual-luciferase reporter assay.

In conclusion, we showed that SNHG22 was downregulated in OS compared with lung metastatic sites. We further found that SNHG22 induced cell cycle arrest in the G1 phase and inhibited the proliferation, migration and invasion of OS cells *in vivo*. This effect occurred mainly through the interaction with miR-4492, which subsequently targeted the transcription of NKIRAS2. SNHG22 also suppressed EMT in OS. In addition, SNHG22 inhibited the migration and proliferation of HUVECs, while SNHG22 silencing showed the opposite effect. Our study has thus demonstrated that SNHG22 was a pivotal regulator of OS and suggested that SNHG22 may be a novel target for treatment of OS.

## MATERIALS AND METHODS

### Bioinformatics analysis

OS patient gene data were obtained from the GEO database (https://www.ncbi.nlm.nih.gov/geo/). The LIMMA package was used for data analysis. The downstream target genes of miR-4492 were predicted using the TargetScan website (http://www.targetscan.org/).

### Cell lines and cell culture

The U2OS, Saos-2, HOS, MG63 cell lines were purchased from the Chinese Academy of Sciences Cell Bank (Shanghai, China). We cultured the cells in DMEM medium containing 10% fetal bovine serum (Thermo Fisher, Waltham, MA, USA), 100 U/mL of penicillin and 100 μg/mL of streptomycin (Invitrogen, Carlsbad, CA, USA) under 5% CO_2_ in a 37°C humidified atmosphere.

### Lentivirus transfection

To construct vectors for SNHG22 upregulation and downregulation, human SNHG22 RNA or short hairpin RNAs for silencing SNHG22 were inserted into the pLVX-Puro vector. U2OS and HOS cells were seeded at 30% density in 6-well plates and transfected with lentivirus in serum-free DMEM medium containing Polybrene (6 μg/mL). Three days after transfection, puromycin was added for cell selection. Unattached cells were removed, and the remaining cells were considered transfected.

### CCK-8 assay

After transfection with the pLVX-vector, pLVX-SNHG22, pLKO.1-vector, or pLKO.1-SNHG22, HOS and U2OS cells were seeded into 96-well plates at a density of 4000cells/well, and 24, 48, 72, and 96 h after transfection, CCK8 reagents (Dojindo Laboratories, Kumamoto, Japan) were added and incubated with the cells for 2 h. Finally, cell viability was measured by absorbance at 450 nm using a microplate reader (Bio-Tek).

### Apoptosis assay

The transfected HOS and U2OS cells were collected and washed with PBS. After incubation with 100 μL of 1× binding buffer containing 5 μL of annexin V-FITC and 10 μL of propidium iodide (PI) for 30 min at room temperature without light, apoptotic transfected cells were counted by flow cytometry(FACSCalibur BD).

### Cell cycle analyses

Transfected U2OS and HOS cells were harvested and washed with PBS, then fixed with 75% ethanol at 4°C overnight. After centrifugation (1,500 × *g*, 3 min), the cells were washed again and resuspended in cold PBS. Suspended cells were incubated with 10 mg/mL RNase and 1 mg/mL PI at 37°C for 30 min without light. Finally, the cell cycle was analyzed by flow cytometry according to the manufacturer’s instructions.

### The qRT-PCR

Total RNA was extracted by TRIzol reagent (Invitrogen) and then reverse-transcribed into cDNA. The qRT-PCR for mRNA detection was performed with the Fast Start Universal SYBR Green Master kit (Roche, Basel, Switzerland). Relative RNA expression levels were measured using the 2-ΔΔCt method by normalizing to GAPDH housekeeping gene expression. microRNA (miRNA) was detected using miRNA Universal SYBR qPCR Master Mix (Vazyme), and U6 was used as a control. Nuclear and cytoplasmic extraction reagents (Thermo Fisher) were used to prepare nuclear and cytoplasmic extracts. Each extract was analyzed by qRT–PCR. The primer sequences are listed as follows: GAPDH, forward: CCACCCATGGCAAATTCCATGGCA, reverse: TCTAGACGGCAGGTCAGGTCCACC; SNHG22, forward: CGAAGGTCTCCTGTGAACCC, reverse: ACATGCTTTGGTGCTGCTTG; U6 forward: CTCGCTTCGGCAGCACA, reverse: AACGCTTCACGAATTTGCGT; E-cadherin, forward: GCTGGACCGAGAGAGTTTCC, reverse: CAAAATCCAAGCCCGTGGTG; ZEB1, forward: GGAGAGGTGACTGGTTGTGG, reverse: GCCACATCAGCAATAGCAGC SNAIL, forward: GCCCCACAGGACTTTGATGA, reverse: CAAAAACCCACGCAGACAGG; N-cadherin, forward: TGGGAAATGGAAACTTGATGGC, reverse: AATCTGCAGGCTCACTGCTC; NKIRAS2, forward: GGGCTGTGCCGGTTGAAAA, reverse: CGTAGATGTCCTCCTGCGTC.

### Transwell assays

U2OS or HOS cells (2×10^5^) were harvested and seeded into the upper chamber of a 24-well Transwell chamber (Costar, Corning, New York, USA) and maintained in serum-free medium. The lower chamber of wells containing Matrigel were incubated in 600 μL of complete medium for 24 h at 37°C. Before staining, the cells were washed and fixed in 4% paraformaldehyde for 30 min. The cells on the upper surface of the chamber were removed with cotton swabs. U2OS or HOS cells which had traversed to the lower surface were stained with 0.1% Crystal Violet (Sigma-Aldrich, St. Louis, MO, USA) for 30 min at 37°C. To quantify invasion, the traversed cells in each chamber were counted in five random fields under a microscope.

### Wound healing assays

We seeded 3×10^6^ transfected U2OS or HOS cells into six-well plates. After growing to 90% confluency, cells in each well were scratched by a 10 μL plastic pipette tip. Images were taken at 0 h and 24 h under a microscope to record cell migration into the wounds.

### ELISA

The supernatant from U2OS or HOS cultures was collected after transfection of cells with lentivirus. The concentration of vascular endothelial growth factor (VEGF) in the supernatants was measured using a human VEGF ELISA Kit (Abcam, ab100662; Cambridge, MA, USA) according to the manufacturer’s instructions.

### Colony-forming assays

U2OS and HOS cells were seeded into six-well plates at a density of 500 cells per well and cultured for 7–10 days. The cells were then washed with PBS three times before fixing with 4% paraformaldehyde. Fixed cells were washed by PBS after staining with 0.1% Crystal Violet for 25 min. The images were taken after the colonies were dried.

### Nuclear and cytoplasmic RNA extraction

Cells were collected and washed once in PBS. The cells were then resuspended in cell fractionation buffer (PARIS Kit, AM1921) for 10 min. Samples were centrifuged for 5 min at 4°C at 500 × *g*. The cytoplasmic fraction was carefully aspirated away from the nuclear pellet and nuclear pellet was lysed in cell disruption buffer. Samples were split for RNA isolation. The lysate was mixed with 2× lysis/binding solution and 100% ethanol. Then the mixture was washed three times and RNA was eluted with elution solution at 95°C and stored at -80°C.

### RNA pull-down assay

The miR-4492 or mimic negative control (NC) labeled with biotin (constructed by and purchased from Sangon) were transfected into U2OS and HOS cell lines. Streptavidin beads were used to pull down the biotin-labeled miRNA from cell lysates following the manufacturer’s instructions [42, 43]. After incubation, elution and decrosslinking, qRT-PCR was performed to examine the expression level of SNHG22.

### RNA immunoprecipitation assay

The MS2bs-MS2bp-based RNA immunoprecipitation (RIP) assay was performed to verify the direct binding between SNHG22 and miR-4492 described in previous research [44]. MS2bs-GFP elements containing SNHG22 were constructed and transfected into HOS cell lines. Cells were harvested 48 h after transfection, then GFP antibody was used to conjugate the MS2bs-GFP elements in the cell extracts. Finally, the miR-4492 expression level was measured by qPCR.

### Dual-luciferase reporter assay

Plasmids containing putative or mutant 3′-UTRs from SNHG22 and NKIRAS2 were constructed and transfected into U2OS or HOS cells with Lipofectamine 3000 (Thermo Fisher Scientific). Cells were collected 24 h after transfection and seeded into 96-well plates. The luciferase activity in the U2OS and HOS cell lines was detected 2 days later after different treatments.

### Spontaneous metastasis xenografts

All animal studies were approved by the Ethics Committee of Xinhua Hospital Affiliated to Shanghai Jiao tong University School of Medicine. Male nude mice (aged 4 weeks, weighing 18–20 g) purchased from the Shanghai Laboratory Animal Center of the Chinese Academy of Sciences (Shanghai, China) were divided into four independent groups with five mice per group. A total of 10^6^ HOS cells transfected with the pLVX-vector, pLVX-SNHG22, pLKO.1-vector or pLKO.1-SNHG22 were subcutaneously injected into the right axilla of each mouse. The tumor volumes were estimated (0.5 × width^2^ × length) by caliper every week. The mice were euthanatized 3 weeks after injection, and the transplanted tumors were carefully dissected and weighed.

### Statistical analysis

Data were expressed as means±SD and all assays were repeated at least three times. The non-parametric Wilcoxon rank-sum test was used to evaluate data from the GEO database. Student’s *t-*test was used for comparisons between groups. Differences were considered significant at P < 0.05.

## Supplementary Material

Supplementary Figure 1
