# Supplementary material for: Characterization of LncRNA SNHG22 as a protector of NKIRAS2 through miR-4492 binding in osteosarcoma
Source: Aging (Albany NY). 2020 Sep 20;12(18):18571–87. doi: 10.18632/aging.103849 (PMC7585113; doi:10.18632/aging.103849)
Supplement: Supplementary Figure 1 [file aging-12-103849-s001..pdf]

SUPPLEMENTARY FIGURE

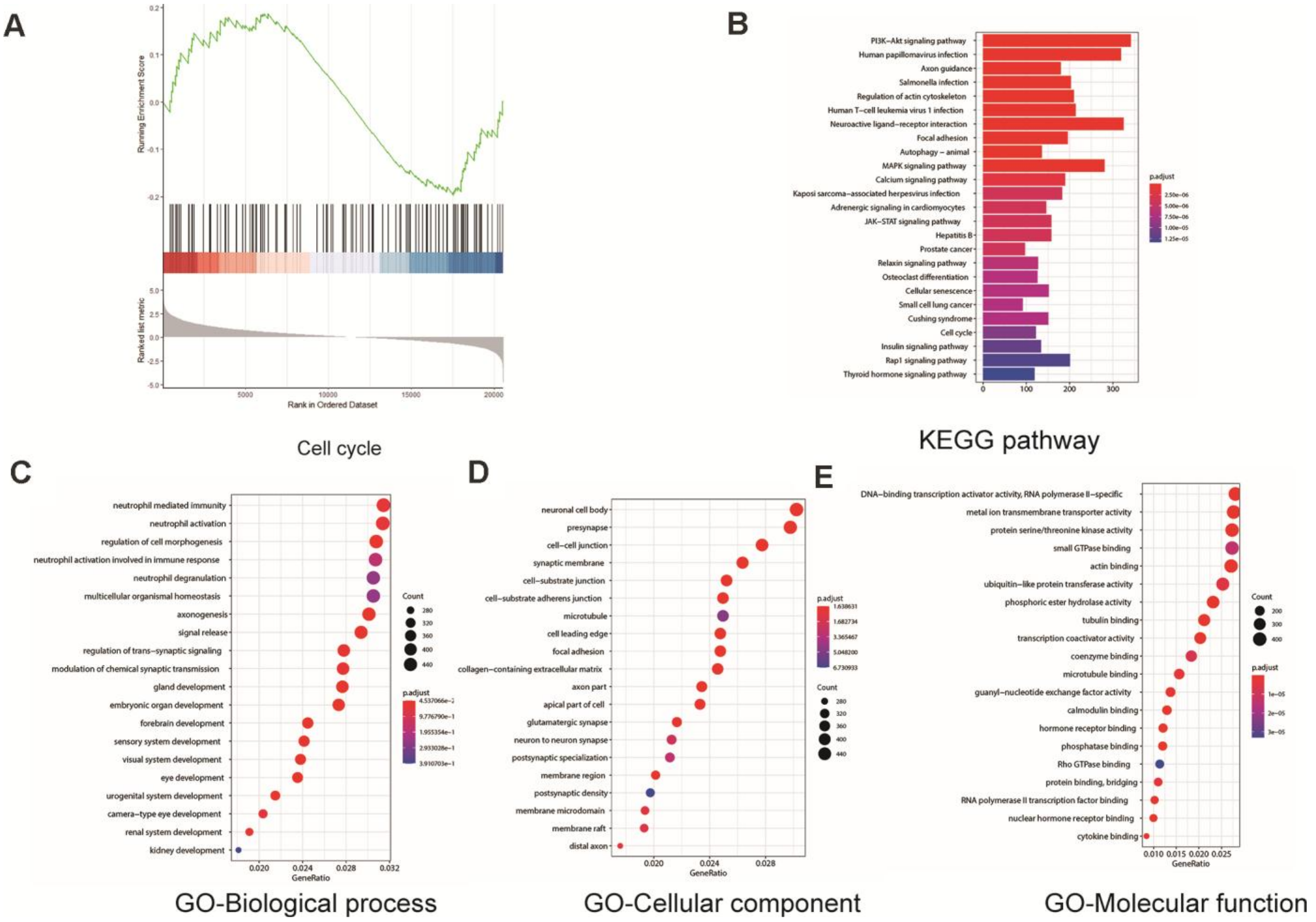

Supplementary Figure 1. Gene Ontology (GO) enrichment analysis, Kyoto Encyclopedia of Genes and Genomes (KEGG) pathway enrichment analysis and Gene Set Enrichment Analysis(GSEA). (**A**) GSEA plot showing that Cell cycle has no significant change. (**B**) KEGG pathways including adjusted p-value as color code. (**C**–**E**) Enriched GO terms including adjusted p-value as color code.
